# T1w/T2w Ratio Identifies the Basolateral Amygdala as a Preferential Target in Autoimmune Limbic Encephalitis

**DOI:** 10.1111/jon.70120

**Published:** 2026-01-22

**Authors:** Rakshit Dadarwal, Andre Dik, Laura Bierhansl, Noëmi Gmahl, Nils C. Landmeyer, Tobias J. Brix, Veronika K. Jaeger, Jochen Bauer, Wilhelm Küker, Heinz Wiendl, Christian E. Elger, Stjepana Kovac, Antje Bischof

**Affiliations:** ^1^ Department of Neurology University Hospital Muenster Muenster Germany; ^2^ Department of Neuroimmunology Center of Neurology University Hospital Bonn Bonn Germany; ^3^ Department of Neurology University Medical Center Schleswig‐Holstein Campus Luebeck and University of Luebeck Luebeck Germany; ^4^ Institute of Medical Informatics University of Muenster Muenster Germany; ^5^ Institute of Epidemiology and Social Medicine University of Muenster Muenster Germany; ^6^ Department of Clinical Radiology University Hospital Muenster Muenster Germany; ^7^ Beta Klinik Bonn Germany

**Keywords:** amygdala nuclei, autoimmune limbic encephalitis, epilepsy, MRI, T1w/T2w ratio

## Abstract

**Background and Purpose:**

The amygdala plays a key role in the pathophysiology of autoimmune limbic encephalitis (ALE), contributing to epileptic seizures and neuropsychiatric symptoms. While no study has examined microstructural changes in individual amygdala nuclei in ALE, we used the T1‐weighted/T2‐weighted (T1w/T2w) ratio to explore amygdalar pathology and its associations with clinical manifestations, including epilepsy and neuropsychiatric symptoms.

**Methods:**

This single‐center study examined 57 patients diagnosed with ALE and 16 healthy controls (HC). Patients underwent a comprehensive assessment that included clinical, electroencephalogram (EEG), magnetic resonance imaging (MRI), and neuropsychological assessments. Patients were stratified by epileptic focus based on long‐term EEG. T1w/T2w ratio and volumetric measures of the amygdala and its nuclei were analyzed and correlated with epileptic focus and neuropsychiatric outcomes.

**Results:**

EEG revealed 26 left temporal, 26 bitemporal, and five right temporal epileptic foci. The T1w/T2w ratio in the left amygdala was markedly reduced in patients with left temporal (*p* = 0.013) and bitemporal (*p* = 0.018) epileptic foci compared to HC. This reduction was most pronounced in the left basolateral complex (*p* = 0.011). Whereas amygdalar volumes were similar between patients and HC, exploratory analyses showed an increased volume of the left lateral nucleus in left temporal ALE (*p* = 0.036). Furthermore, we found no correlations between MRI measures and neuropsychiatric scores.

**Conclusion:**

Our findings indicate that the basolateral complex of the amygdala is preferentially affected in ALE, suggesting a region‐specific vulnerability to autoimmune‐mediated inflammation. T1w/T2w ratio alterations reflect the epileptogenic focus and may serve as a clinically accessible, noninvasive biomarker for early diagnosis and treatment monitoring in ALE.

## 1 Introduction

Autoimmune limbic encephalitis (ALE) is an autoimmune‐mediated inflammatory disease of the central nervous system targeting key structures of the limbic system, most notably the hippocampus and amygdala. Early diagnosis of ALE is crucial for individual treatment stratification and prevention of complications to improve the long‐term prognosis for affected individuals. Diagnosis of ALE relies on a combination of clinical symptoms, imaging, cerebrospinal fluid findings, and antibody testing [1, 2, 3]. The manifestations of ALE are highly variable, including cognitive deficits, seizures, depression, and anxiety [1, 2]. Due to their nonspecific nature, these clinical manifestations are frequently under‐reported or misdiagnosed [2, 3]. While antibody testing is crucial for identifying immunological subtypes of ALE and confirming the diagnosis, it can be negative, including false‐negative results [4, 5].

Neuroimaging is crucial for ALE diagnosis and monitoring. Magnetic resonance imaging (MRI) is the primary diagnostic tool, and the most prominent MRI changes are visually observed in mesial temporal lobe structures, including the amygdala and hippocampus [6, 7]. Unilateral or bilateral T2‐weighted (T2w)/fluid‐attenuated inversion recovery (FLAIR) hyperintense signal changes in the medial temporal lobe are a hallmark of ALE [8]. These are included in the Graus diagnostic criteria as a key diagnostic tool when antibodies are absent [7]. In the early stages, amygdala swelling and T2w/FLAIR hyperintensity have been linked to neuroinflammation, which may be followed by neurodegeneration presenting as atrophy on MRI [9, 10, 11, 12]. However, current methods of assessing these changes in the amygdala rely on visual inspection, making the assessment subjective. Therefore, there is an urgent need for more objective, quantitative MRI biomarkers to detect changes in tissue integrity throughout the disease course.

The T1‐weighted (T1w)/T2w ratio is a semiquantitative measure of relative myelin content. It can delineate cortical areas and enhance myelin contrast‐to‐noise ratio [13]. The use of the T1w/T2w ratio has been extended from neurological diseases of cerebral gray matter to white matter, including multiple sclerosis [14, 15]. Histopathological studies suggest that the T1w/T2w ratio reflects various aspects of tissue microstructure, including myelin integrity, axon and dendrite density, and iron content [16]. The widespread availability of T1w and T2w sequences as part of routine standard MRI protocols is a significant advantage, enabling rapid translation into clinical practice. Moreover, automated processing eliminates variability associated with subjective visual assessment.

The amygdala is a key structure of the limbic system that processes emotions and generates appropriate behavioral responses to stimuli. Structural and functional alterations of the amygdala have been linked to various mental health conditions, including mood disorders, anxiety, and psychosis [17]. Furthermore, amygdala pathology has been associated with epileptogenesis and epilepsy, which are prominent clinical features in ALE [17]. While prior research has predominantly focused on hippocampal involvement, the amygdala has received comparatively little attention, partly due to technological challenges in precisely delineating the anatomical boundaries of this small gray matter structure. However, recent advances in neuroimaging now allow for precise parcellation of the amygdala nuclei and their microstructural alterations. Emerging evidence suggests a critical role of the amygdala in epileptogenesis and cognitive dysfunction in temporal lobe and idiopathic generalized epilepsy, and for the risk of sudden unexpected death in epilepsy [18, 19]. Additionally, a recent study found a reduced T1w/T2w ratio in the amygdala in anti‐NMDA‐receptor encephalitis, while no correlation with neuropsychological testing was reported [15]. Furthermore, amygdala nuclei involvement may aid in the differential diagnosis of disorders underlying mesial temporal lobe epilepsy [19]. However, a comprehensive evaluation of pathological changes in the amygdala nuclei in ALE using advanced microstructural MRI measures, such as the T1w/T2w ratio, is lacking.

In this study, we investigated changes in the T1w/T2w ratio and volume of the amygdala and its nuclei in ALE patients compared to healthy controls (HC). Moreover, we examined potential clinicoradiological correlations of these measures with clinical features in ALE, including epileptic seizures and neuropsychiatric symptoms, such as depression, anxiety, and facial emotion recognition.

## 2 Methods

### 2.1 Study Cohort

Patients were recruited retrospectively and prospectively from the Epilepsy Center of the University Hospital of Muenster, Germany, according to the following criteria: (1) a diagnosis of ALE according to the Graus criteria [5]; (2) age >18 years; (3) ability to remain in an MRI scanner for 1 h or existing MR scan acquired at our hospital using the protocol detailed below. Patients were excluded if they met any of the following criteria: (1) other neuropsychiatric disorders; (2) contraindications for MRI scanning; or (3) inability to provide written informed consent. An additional subset of HC was scanned using the same MRI protocol. Patients were stratified into three subgroups based on electroencephalogram (EEG) findings, including seizures, epileptic discharges, and focal slowing, according to the laterality of seizure location. Written informed consent was obtained from all participants in the study. The study was approved by the internal review board of the University of Muenster (ethics vote no 2013‐350‐f‐S).

### 2.2 MRI Data Acquisition

All MR images were acquired on the same clinical 3T scanner (Philips Medical Systems, Best, The Netherlands), which underwent two major software upgrades during the study period. The protocol included (1) a transversal 3D T1w Turbo Field Echo (TFE) sequence with the following parameters: resolution = 0.63×0.63 mm^2^, repetition time (TR) = 10.1 ms, echo time (TE) = 5.7 ms, flip angle (FA) = 8°, field of view (FOV) = 220×220 mm^2^, matrix size = 352×352, and slice thickness = 1 mm and (2) a high‐resolution paracoronal 2D T2w Turbo Spin Echo (TSE) sequence with temporal angulation to the hippocampus with the following parameters: resolution = 0.23×0.23 mm^2^, TR = 5197.7 ms, TE = 118 ms, number of averages = 2, FOV = 200×200 mm^2^, matrix size = 880×880, slice thickness = 2 mm, and slice gap = 0.2 mm.

### 2.3 MRI Data Analysis

**FIGURE 1:**
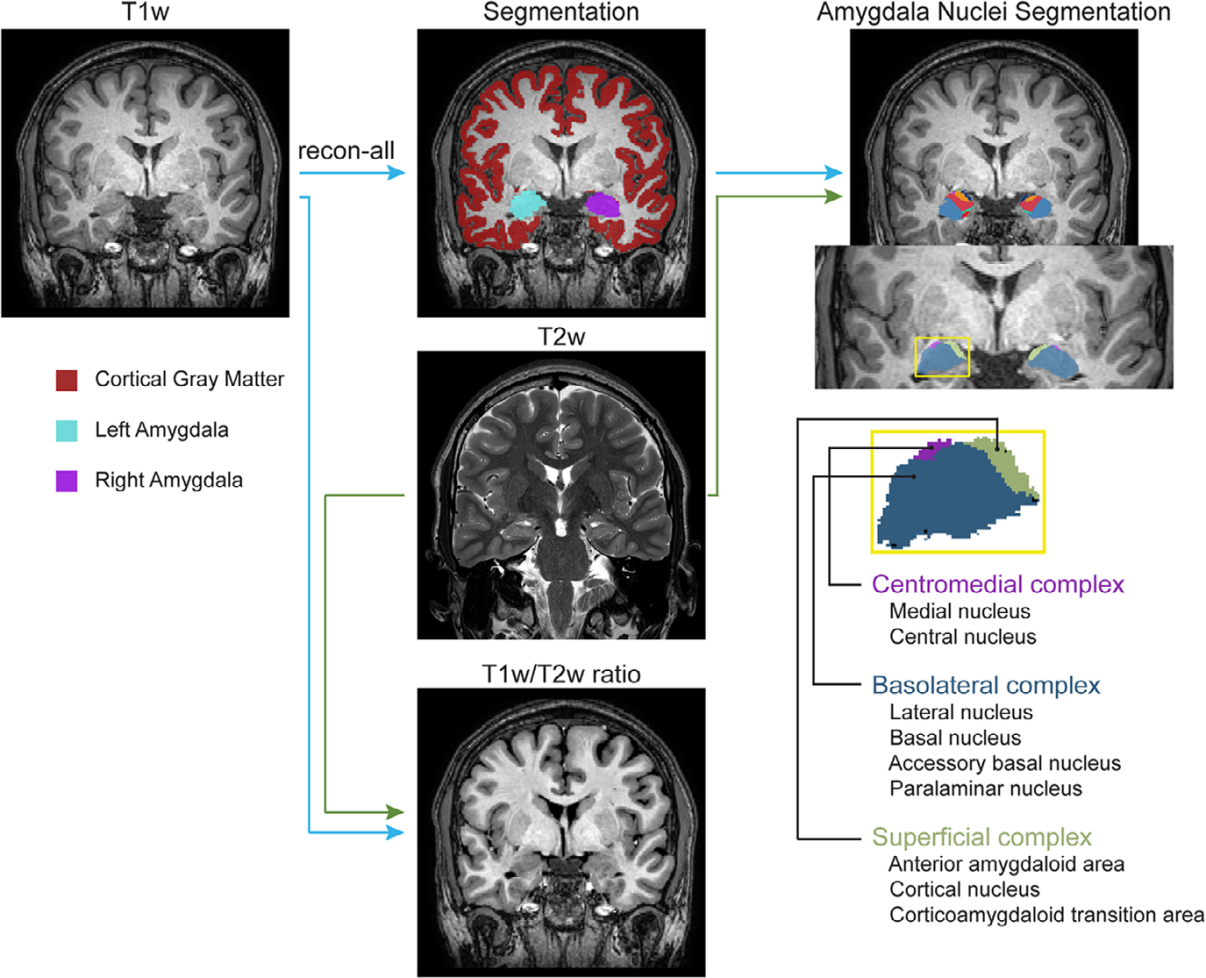
MRI data processing pipeline. T1w images were preprocessed with FreeSurfer recon‐all, and the cortical gray matter and left and right amygdala segmentations were derived. High‐resolution T2w images were used as an additional input to segment the amygdala nuclei. The segmented nine amygdala nuclei were grouped into three complexes: basolateral complex (lateral nucleus, basal nucleus, accessory basal nucleus, and paralaminar nucleus), superficial complex (anterior amygdaloid area, cortical nucleus, and corticoamygdaloid transition area), and centromedial complex (medial nucleus and central nucleus). The T1w/T2w ratio was created after aligning the bias field‐corrected T1w and T2w images.

All MRI analyses were performed blinded to the clinical data. Quality assessment of T1w and T2w images was performed using MRIQC [20]. Cortical gray matter and amygdala masks were obtained from T1w images using FreeSurfer software (v7.4.1, Athinoula A. Martinos Center for Biomedical Imaging, Charlestown, MA, USA, https://surfer.nmr.mgh.harvard.edu/; Figure 1) [21]. Amygdalar nuclei were segmented from T1w and high‐resolution T2w images using FreeSurfer [22].

For T1w/T2w ratio calculations, T1w and T2w images were bias‐corrected using ANTs [23]. T2w images were coregistered to the T1w image using FSL FLIRT [24]. A binarized cerebral cortex mask was computed, and the medians of the cerebral cortex mask for T1w and T2w images were divided to calculate the scaling factor (s). The standardized T1w/T2w ratio (sT1w/T2w ratio) was then computed using the following equation as described in Misaki et al. [25]: sT1w/T2w ratio = (T1w—sT2w)/ (T1w + sT2w).

For simplicity, we denote the “sT1w/T2w ratio” as “T1w/T2w ratio” throughout this manuscript. The segmented nine amygdala nuclei were grouped into three major complexes: basolateral complex (lateral nucleus, basal nucleus, accessory basal nucleus, and paralaminar nucleus), superficial complex (anterior amygdaloid area, cortical nucleus, and corticoamygdaloid transition area), and centromedial complex (medial nucleus and central nucleus; Figure 1) [26]. Estimated amygdala volumes were normalized for head size using the total intracranial volume.

### 2.4 Neuropsychiatric Assessment

Neuropsychiatric symptoms were assessed using standardized clinical test scores. To evaluate mood, the Hospital Anxiety and Depression Scale (HADS) questionnaire [27] or the Beck Depression Inventory‐II (BDI‐II) [28] was used. Specifically, the HADS questionnaire was designed to identify individuals with anxiety disorders and depression, particularly among patients with somatic and psychiatric illnesses. The facial emotion recognition test (FERT), a subset of the mini‐SEA score [29], was used to assess the ability to recognize and interpret facial expressions.

### 2.5 Statistical Analysis

All statistical analyses were performed using R (v4.3.3, R Foundation for Statistical Computing, Vienna, Austria, https://www.r‐project.org), JMP Student Edition (v18.2.2, JMP Statistical Discovery LLC, Cary, NC, USA, https://www.jmp.com), and Python (v3.13.2, Python Software Foundation, Beaverton, OR, USA, https://www.python.org). Comparisons of demographic data between HC and patients were performed using Chi‐square or Student's *t*‐tests, as appropriate. Neuropsychiatric measures were transformed to z‐scores. Group differences in MRI measures were assessed using a linear regression model, adjusting for age, sex, and MRI scanner software version. Using a residual−residual correlation, Pearson's correlation coefficient was calculated to evaluate associations between the T1w/T2w ratio and neuropsychiatric symptoms. The significance level was set to *p*<0.05. The Benjamini−Hochberg false discovery rate (FDR) method was applied to adjust for multiple comparisons.

## 3 Results

### 3.1 Demographic and Clinical Characteristics

From July 2016 to August 2023, 57 patients with ALE and 16 HC (median age 37, seven females) were included in the study. Demographic and clinical data at the time of MRI acquisition are summarized in Table 1. Using available long‐term EEG (LT‐EEG) monitoring findings acquired before or at the time of MRI, 26 patients had left temporal (LT ALE, median age 47), five had right temporal (RT ALE, median age 61), and 26 had bitemporal (BT ALE, median age 51) epileptic ictal or interictal activity. Thirty‐eight (67%) patients had bilateral tonic–clonic seizures in addition to focal seizures. This proportion was even higher in the subgroup of right temporal ALE, where 4/5 (80%) patients had tonic–clonic seizures. Notably, 42/52 (81%) patients with LT or BT ALE had an LT‐EEG evaluation at the time of MRI acquisition, with the LT‐EEG recording starting or ending <1 day before or after the MRI acquisition. In 9/42 (21%) patients, at least one seizure was registered on the LT‐EEG recording over the 3‐ to 4‐day monitoring period. Seropositive and seronegative ALE cases were evenly distributed between the LT and BT groups, while all five RT patients were seropositive. Neuropsychiatric assessments were available in a subset of ALE patients. BDI II and HADS scores assessing anxiety and depression were available in 32 (LT/BT: 17/15) and 42 (LT/BT: 20/22) ALE patients, respectively. The FERT score assessing facial emotion recognition (maximum score 15) was available in 25 (LT/BT: 14/11) patients. Most patients (56 patients, 98%) were prescribed anti‐seizure medication (ASM) after ALE diagnosis. Among them, 26 (46%) were treated with one or two drugs, while 30 (53%) received more than two ASMs. Overall, 26 (46%) patients were on antidepressants. At the time of the MRI acquisition, 22 (38.6%) of the ALE patients were therapy naïve. First‐line treatment included intravenous methylprednisolone or plasma exchange, while second‐line treatment included azathioprine, mycophenolate mofetil, or rituximab. Immunomodulatory treatments are specified in Table 1.

**TABLE 1:** Details of demographic, clinical, and diagnostic characteristics of HC and ALE.

Characteristic	HC (*n* = 16)	ALE (*n* = 57)	*p*
Demographics			
Gender, female, *n* (%)	7 (43.7)	27 (47.4)	1.000
Age at onset, median, years (IQR)	31 (21.5)	47 (27.5)	**0.003**
Follow‐up time, median, months (IQR)		37 (29)	
Diagnostic testing prior to therapy			
CSF, *n* (%)		56 (98)	
Lymphocytes >4/µL		3 (5.3)	
Increased protein level		11 (19.3)	
OCB			
Type 1		44 (77.2)	
Type 2		9 (15.8)	
Type 3		3 (5.3)	
Antibody present, *n* (%)		25 (43.9)	
CASPR2		7 (12.3)	
GAD65		7 (12.3)	
LGI1		3 (5.3)	
NMDAR		3 (5.3)	
Other, name		1xMa2/Ta; 2xNeuropil; 2xZIC4	
Clinical features at the time of MRI acquisition			
EEG features (*n* %)			
Long‐term EEG available		45 (86.5)	
Seizure during EEG (ictal patterns)		10 (22.2)	
Interictal patterns during EEG		19 (42.2)	
Treatment (*n* %)			
Naïve		22 (38.6)	
First‐line (active^a^)		4 (7.0)	
First‐line (past^a^)		18 (31.6)	
Second‐line (active^b^)		6 (10.5)	
Second‐line (past^b^)		7 (12.3)	

*Note*: Significant *p*‐values are bolded.

Abbreviations: ALE, autoimmune limbic encephalitis; CSF, cerebrospinal fluid; EEG, electroencephalography; HC, healthy controls; IQR, interquartile range; *n*, number of individuals; OCB, oligoclonal bands.

^a^ Active first‐line treatment was defined as therapy administered within the last 6 weeks prior to MRI; past first‐line treatment was defined as treatment administered longer than 6 weeks prior to MRI.

^b^ Active second‐line treatment was defined as treatment administered within the last 6 months prior to MRI. Past second‐line treatment was defined as treatment administered longer than 6 months prior to MRI.

### 3.2 T1w/T2w Ratio of the Amygdala

**FIGURE 2:**
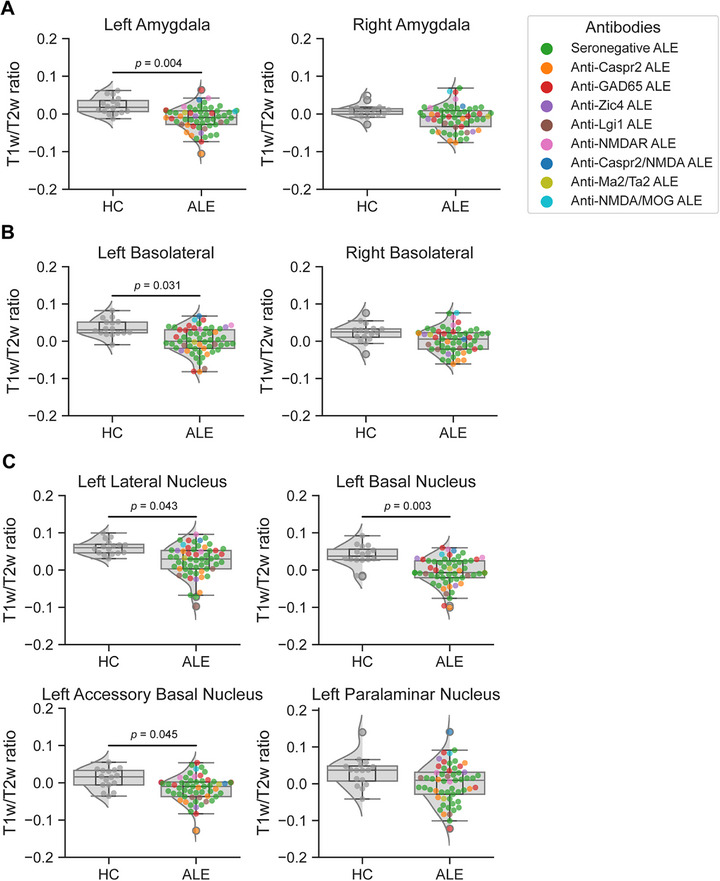
T1w/T2w ratio of the amygdala in HC and patients with ALE. (A) Comparison of left and right amygdala T1w/T2w ratio between HC and patients with ALE. (B) Comparison of the T1w/T2w ratio of the left and right basolateral complex between healthy controls (HC) and patients with autoimmune limbic encephalitis (ALE). (C) Comparison of basolateral complex nuclei T1w/T2w ratio in the left amygdala between HC and patients with ALE. The basolateral complex of the amygdala includes lateral, basal, accessory basal, and paralaminar nuclei. *p*‐Value*s* were adjusted for multiple comparisons using the false‐discovery rate method. Abbreviations: MOG, myelin oligodendrocyte glycoprotein; NMDA, N‐methyl‐D‐aspartate.

The T1w/T2w ratio in the left amygdala was markedly reduced in patients with ALE (*p*(FDR) = 0.004; 95% confidence interval [CI]: {0.010; 0.044}) compared to HC (Figure 2A). When examining the basolateral, superficial, and centromedial amygdala complexes, respectively, each stratified by right and left side, the T1w/T2w ratio in the left basolateral complex was decreased in patients with ALE compared to HC (*p*(FDR) = 0.031; 95% CI: {0.009; 0.047}; Figure 2B). The superficial (*p*(FDR) = 0.145; 95% CI: {−0.001; 0.046}) and centromedial (*p*(FDR) = 0.145; 95% CI: {−0.003; 0.062}) complexes showed no differences in the T1w/T2w ratio between patients with ALE and HC. Further analysis of the left basolateral complex showed a decreased T1w/T2w ratio in patients with ALE in the lateral nucleus (*p*(FDR) = 0.043; 95% CI: {0.004; 0.048}), basal nucleus (*p*(FDR) = 0.003; 95% CI: {0.007; 0.016}), and accessory basal nucleus (*p*(FDR) = 0.045; 95% CI: {0.002; 0.038}) when compared to HC (Figure 2C).

**FIGURE 3:**
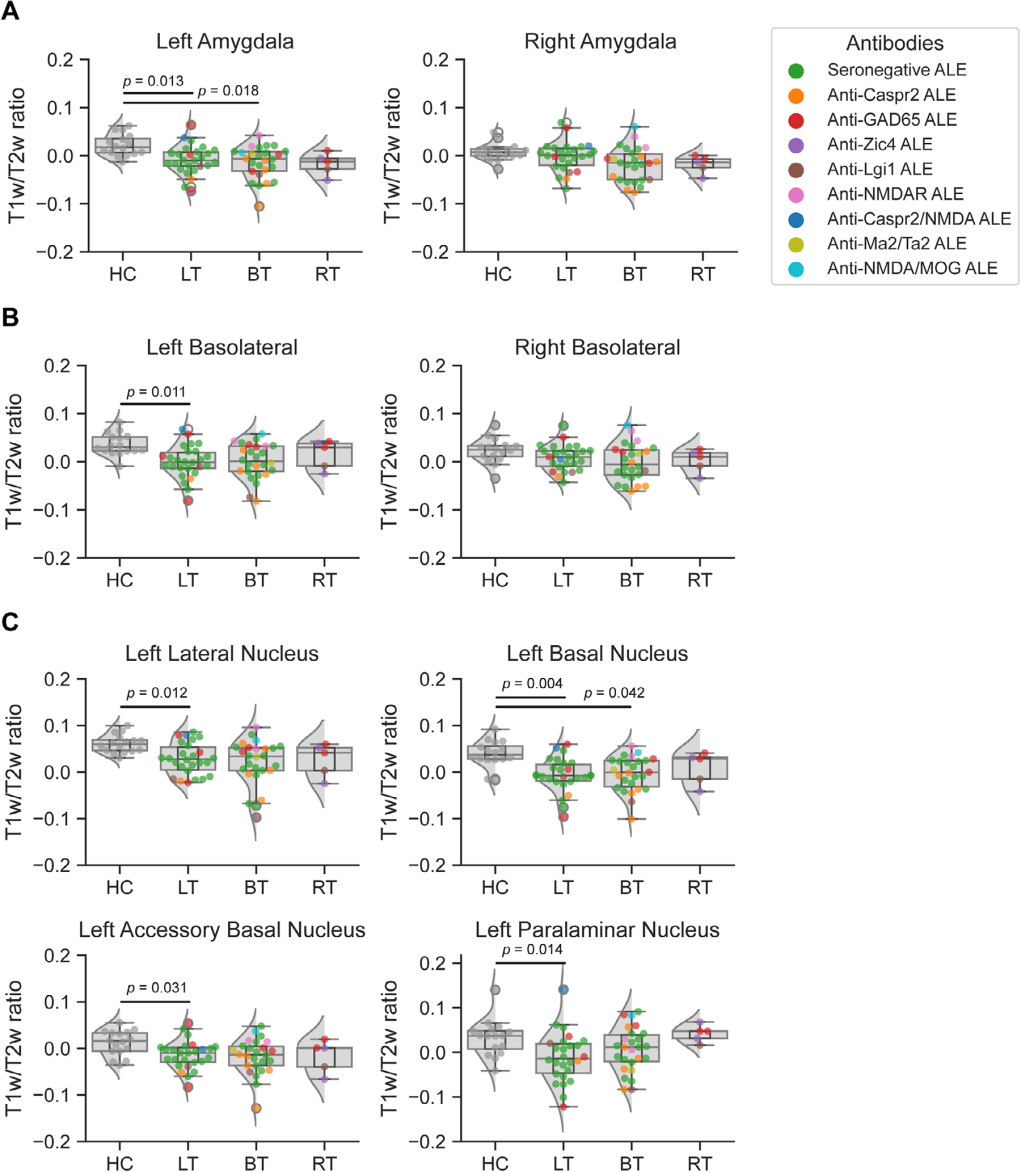
T1w/T2w ratio of the amygdala in healthy controls (HC) and patients with left temporal (LT), bitemporal (BT), and right temporal (RT) autoimmune limbic encephalitis (ALE). (A) Comparison of left and right amygdala T1w/T2w ratio between HC and patients with LT, BT, and RT ALE. (B) Comparison of the T1w/T2w ratio of the left and right basolateral complex between HC and patients with LT, BT, and RT ALE. (C) Comparison of basolateral complex nuclei T1w/T2w ratio in the left amygdala between HC and patients with LT, BT, and RT ALE. *p*‐Value*s* were adjusted for multiple comparisons using the false‐discovery rate method. Patients with RT ALE were not included in the final analyses due to the small sample size, but analyses are shown for illustration. Abbreviations: MOG, myelin oligodendrocyte glycoprotein; NMDA, N‐methyl‐D‐aspartate.

Moreover, subgroup analyses showed that the T1w/T2w ratio in the left amygdala was markedly reduced in patients with LT (*p*(FDR) = 0.013; 95% CI: {0.008; 0.045}) and BT (*p*(FDR) = 0.018; 95% CI: {0.008; 0.051}) with ALE compared to HC (Figure 3A). By contrast, the right amygdala in LT and BT ALE showed no difference compared to HC. When examining the amygdala complexes, the T1w/T2w ratio in the left basolateral complex was decreased in LT ALE compared to HC (*p*(FDR) = 0.011; 95% CI: {0.014; 0.056}; Figure 3B). The superficial (LT = *p*(FDR) = 0.222; 95% CI: {−0.006; 0.051}) and centromedial (LT = *p*(FDR) = 0.185; 95% CI: {−0.002; 0.069}) complexes showed no differences in the T1w/T2w ratio in LT and BT ALE, respectively, compared to HC.

**FIGURE 4:**
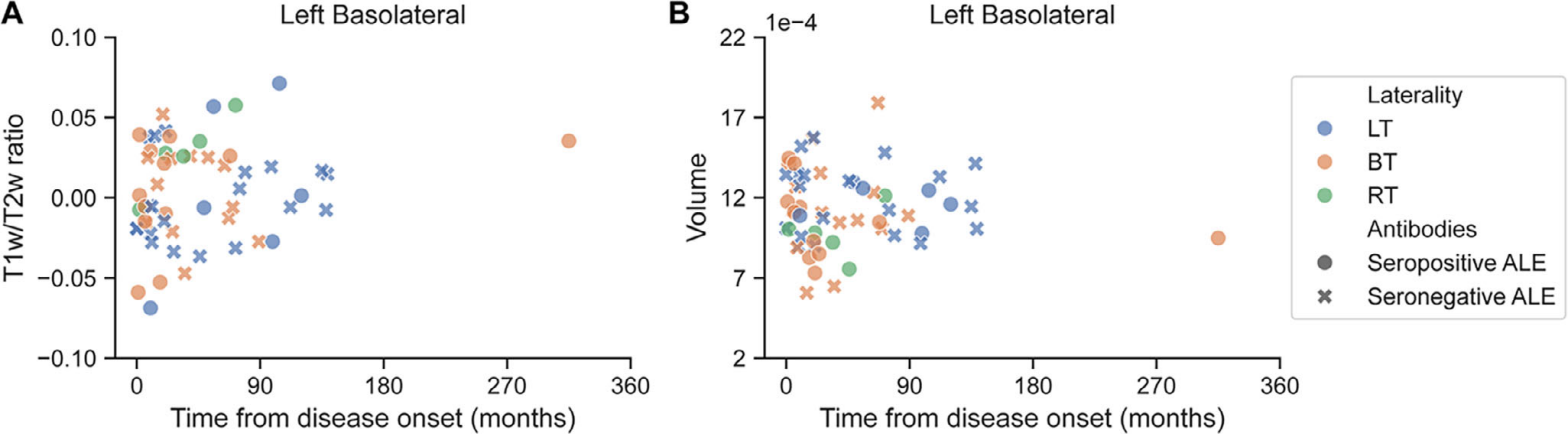
T1w/T2w ratio and volume of the left basolateral complex relative to the time from disease onset. (A) T1w/T2w ratio in the left basolateral complex, adjusted for age, sex, and MRI scanner software versions relative to the time from disease onset. (B) Volume of the left basolateral complex, adjusted for age, sex, and MRI scanner software versions, relative to the time from disease onset. Abbreviations: ALE, autoimmune limbic encephalitis; BT, bitemporal; LT, left temporal; RT, right temporal.

Further analysis of the left basolateral complex showed a lower T1w/T2w ratio in patients with LT ALE in all four nuclei when compared to HC, including the lateral nucleus (*p*(FDR) = 0.012; 95% CI: {−0.009; 0.050}), basal nucleus (*p*(FDR) = 0.004; 95% CI: {0.018; 0.065}), accessory basal nucleus (*p*(FDR) = 0.031; 95% CI:{0.002; 0.042}), and paralaminar nucleus (*p*(FDR) = 0.014; 95% CI: {0.012; 0.083}; Figure 3C). Patients with BT ALE had a reduced T1w/T2w ratio in the basal nucleus compared to HC (*p*(FDR) = 0.042; 95% CI: {0.008; 0.057}). Figure 4A illustrates the T1w/T2w ratio values in the left basolateral complex relative to the time from disease onset.

In order to exclude a potential bias from immunotherapy, we performed a sensitivity analysis in the subgroup of treatment‐naïve ALE patients (*n* = 20). Similar to the main findings, we observed a decrease in the T1w/T2w ratio in the left amygdala (*p*(FDR) = 0.001; 95% CI: {0.017; 0.063}) and the left basolateral complex (*p*(FDR) = 0.002; 95% CI: {0.023; 0.071}). Due to the limited sample size, further analyses stratified by treatment strategy (e.g., recent steroid pulse) were not conducted.

To exclude a potential effect of immediate seizures at the time of MRI acquisition on the T1w/T2w ratio results, we performed a sensitivity analysis in the subset of patients who were seizure‐free (SF‐ALE) at the time of MRI as confirmed by simultaneous or near‐simultaneous LT‐EEG. We identified 42/57 (74%) BT and LT ALE patients in whom LT‐EEG was available at the time of MRI, with a median of 0 (mean = 0.5; IQR = 1, range 3) days from MRI. Of these, 30/42 (77%) were seizure‐free. When repeating the main analyses in this subgroup, results remained similar with almost identical effect sizes, though significance was less pronounced due to the reduction in sample size: the Tw/T2w ratio was markedly reduced in left amygdala (*p*(FDR) = 0.01; 95% CI: {0.008; 0.050}) in LT and BT SF‐ALE patients compared to HC. The T1w/T2w ratio in the left basolateral complex demonstrated the same trend in SF‐ALE patients compared to HC (*p*(FDR) = 0.059; 95% CI: {0.005; 0.051}). Further analysis of the left basolateral complex showed a decreased T1w/T2w ratio in SF‐ALE patients in the lateral nucleus (*p*(FDR) = 0.015; 95% CI: {0.009; 0.055}) and basal nucleus (*p*(FDR) = 0.015; 95% CI: {0.012; 0.062}) when compared to HC. Using the Hedges’ *g*, which measures the standardized difference between the means of two groups, we confirmed that the reductions in the T1w/T2w ratio in patients compared to controls were almost identical in the SF‐ALE and ALE groups.

### 3.3 Volumetric Analysis of the Amygdala

**FIGURE 5:**
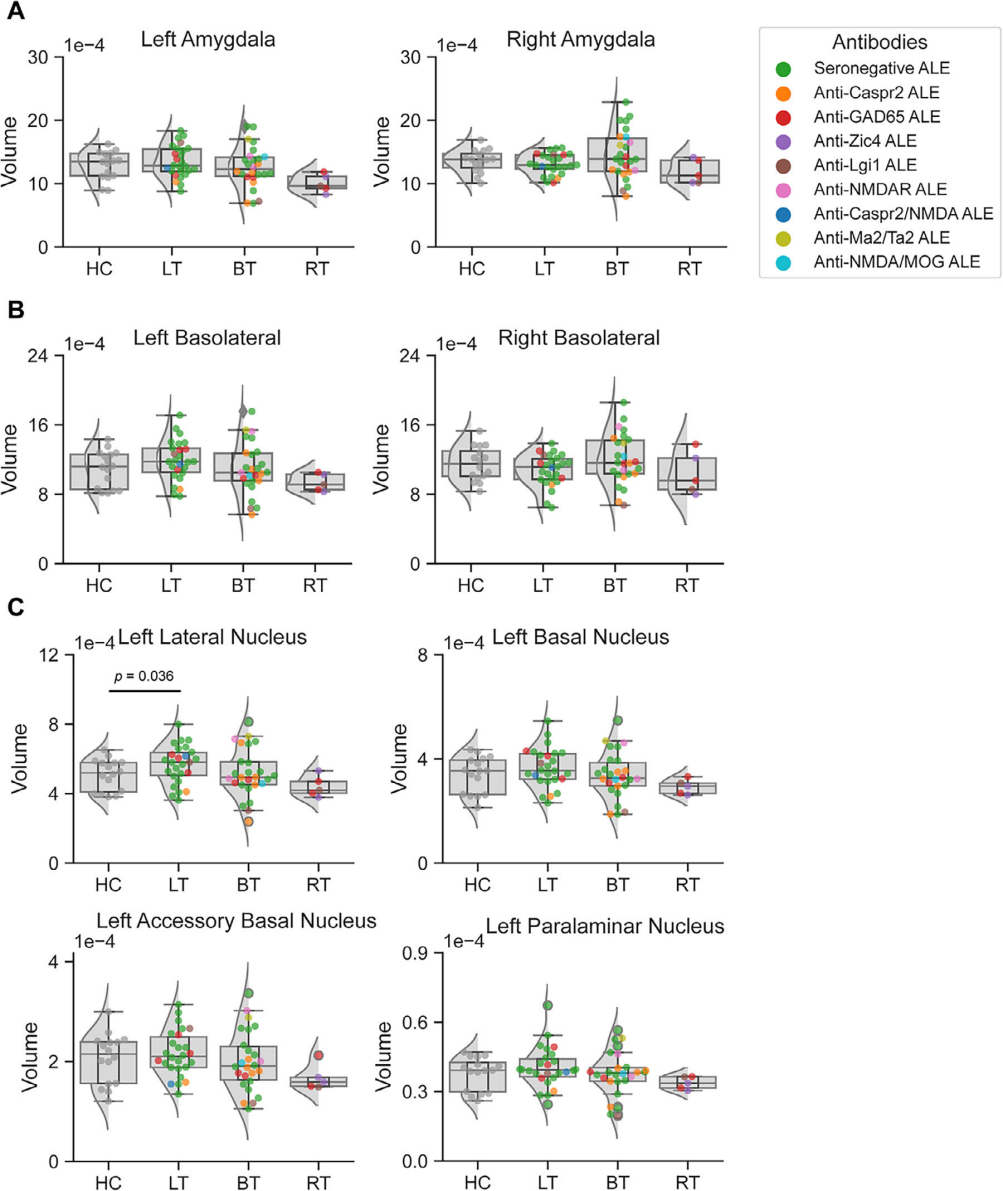
Normalized amygdala volumes in healthy controls (HC) and patients with left temporal (LT), bitemporal (BT), and right temporal (RT) autoimmune limbic encephalitis (ALE). (A) Comparison of left and right amygdala volumes between HC and patients with LT, and BT ALE. (B) Comparison of the volumes of the basolateral complex in the left and right hemispheres between HC and patients with LT, and BT ALE. (C) Comparison of basolateral complex nuclei volumes in the left amygdala between HC and patients with LT, and BT ALE. The basolateral complex includes the lateral, basal, accessory basal, and paralaminar nuclei. The amygdala volumes were normalized to total intracranial volume, yielding unitless values. *p*‐Value*s* were adjusted for multiple comparisons using the false‐discovery rate method. Abbreviations: MOG, myelin oligodendrocyte glycoprotein; NMDA, N‐methyl‐D‐aspartate.

The left and right amygdala volumes in LT and BT ALE patients were similar to HC (Figure 5A). From visual inspection, seropositive and seronegative ALE cases were evenly distributed between the LT and BT groups (Figure 5). In exploratory analyses, we assessed whether there were any differences in the volume of the amygdala nuclei in patients with LT and BT ALE compared to HC (Figure 5B). Interestingly, the volume of the left lateral nucleus within the basolateral complex was increased in the subgroup of patients with LT ALE compared to HC (*p*(FDR) = 0.036; 95% CI: {−15e‐05; −2e‐05}; Figure 5C). Furthermore, a similar trend was noted in this patient subgroup in the basal, accessory basal, and paralaminar nuclei (Figure 5C). Importantly, the increased volume was present irrespective of disease duration, indicating that it might persist over time in at least some patients. Figure 4B illustrates the volume of the left basolateral complex in relation to the time from disease onset.

### 3.4 Correlations Between T1w/T2w Ratio and Neuropsychiatric Symptoms

Neuropsychiatric scores assessed in this study did not correlate with microstructural or volumetric MRI measures. The Pearson correlation coefficient of the T1w/T2w ratio of the left and right amygdala with the FERT facial emotion recognition score was *r* = 0.2 (*p* = 0.287; 95% CI: {−0.6; 0.2}) and *r* = 0.2 (*p* = 0.217; 95% CI: {−0.1; 0.6}), respectively. There was no correlation between the T1w/T2w ratio of the left and right amygdala and the depression score (*r* = −0.1, *p* = 0.514; 95% CI: {−0.4; 0.2} and *r* = 0.2 (*p* = 0.217; 95% CI: {−0.1; 0.6}, respectively). There was no correlation of the T1w/T2w ratio of the left and right amygdala with the anxiety score (*r* = −0.1, *p* = 0.510; 95% CI: {−0.4; 0.2} and *r* = −0.1, *p* = 0.588; 95% CI: {−0.4; 0.3}, respectively). There were no significant correlations between neuropsychiatric scores and T1w/T2w ratio changes in the left basolateral nuclei complex or right amygdala, nor were there any correlations between neuropsychiatric scores and amygdala volumes (data not shown).

## 4 Discussion

This in vivo study demonstrates that the basolateral complex of the amygdala is preferentially affected in autoimmune encephalitis, suggesting a region‐specific vulnerability to autoimmune‐mediated inflammation. T1w/T2w ratio changes were most pronounced in the basolateral complex of the amygdala, which is known to play a role in epileptogenesis, and correlated with the epileptogenic focus. Notably, we observed a pronounced asymmetry in amygdala pathology, with a predominant impact on the left side: most patients exhibited either left or bilateral involvement based on LT‐EEG monitoring findings. Consistent with these LT‐EEG findings, microstructural (T1w/T2w ratio) and volumetric alterations were lateralized to the left side when compared to HC, particularly in patients with left‐sided ALE from LT‐EEG. Importantly, our results were confirmed in patients without seizure activity on LT‐EEG monitoring at the time of MRI. Exploratory subgroup analyses demonstrated an increased volume of the left lateral nucleus in patients with LT ALE compared to HC. This study provides evidence that the T1w/T2w ratio obtained from standard clinical epilepsy protocols is sensitive to clinically relevant microstructural alterations in the amygdala and its nuclei in ALE patients. Our findings underscore its potential as a noninvasive biomarker for diagnosis and monitoring in ALE.

Most cases of ALE with mesiotemporal lobe involvement on T2w/FLAIR images include amygdalar abnormalities, suggesting alterations to the underlying microstructure [7, 30]. However, neuroimaging studies assessing these changes are sparse. A recent study found T1w/T2w ratio reductions in anti‐NMDA‐receptor encephalitis patients in deep gray matter structures, including the amygdala [15]. Building on these findings, our results demonstrate that T1w/T2w ratio changes in the amygdala can be found in a broad spectrum of antibody‐positive and seronegative ALE cases. For the first time, we investigated microstructural changes of individual amygdala nuclei in ALE using a novel parcellation algorithm. Interestingly, the observed T1w/T2w ratio changes were most severe in the basolateral complex of the amygdala, suggesting a preferential anatomical involvement in ALE. The study by Ballerini et al. [31] compared amygdala nuclei volumes in patients with temporal lobe epilepsy from hippocampal sclerosis (TLE‐HS) to nonlesional MRI‐negative temporal lobe epilepsy cases and found distinct anatomical patterns between groups. TLE‐HS patients exhibited pronounced atrophy in the basolateral amygdala, whereas MRI‐negative patients showed prominent swelling of the medial nucleus. The mechanisms underlying these distinct anatomical patterns remain elusive. In our study, the anatomical pattern was similar to that of the TLE‐HS group. The basolateral complex is the primary amygdalar structure engaged in reciprocal connections with the hippocampus. This could explain its preferential involvement in disorders with marked hippocampal pathology, such as TLE‐HS or ALE [32]. Another important anatomical distinction between the basolateral and central amygdalar complex is their cytoarchitecture: the basolateral complex is cortex‐like and composed predominantly of glutamatergic projection neurons. By contrast, the centromedial complex of the amygdala is mainly GABAergic and resembles striatal organization [33]. These differences raise the possibility that the underlying pathophysiological mechanisms, such as autoimmune processes in ALE, preferentially target neuronal populations with specific cytoarchitectural properties.

While there is a large body of evidence assessing the relationship of epilepsy and seizures with hippocampal structures, the role of the amygdala has received little attention [34]. This is particularly intriguing as the amygdala seems to play an equally important role in the pathophysiology of the generation of epileptic potentials via glutamatergic and GABAergic pathways, resulting in hyperexcitability [34]. Histopathological studies in patients with TLE demonstrated that the most severe damage from epileptic activity occurs in the basolateral complex of the amygdala. These findings were corroborated by work in nonhuman primates and kindling studies in rat models [34]. In our study, all patients underwent LT‐EEG monitoring to determine the epileptogenic focus. We found that most patients had either a left or bitemporal epileptogenic focus, which was associated with a decrease in the T1w/T2w ratio in all regions of the basolateral complex, indicating severe microstructural alterations. Sensitivity analyses excluding patients with seizures on LT‐EEG monitoring at the time of MRI confirmed our results. This suggests that the observed T1w/T2w ratio alterations reflect persistent structural changes, for example, from remodeling following damage or neurogenesis [35]. However, due to the cross‐sectional nature of our study, it remains unclear whether these changes occurred as a result of the initial immunological attack or from damage following repetitive epileptic activity over time.

Our study highlights that the microstructural integrity is most impacted when the primary epileptogenicity side is lateralized to the left temporal lobe. Most patients had pathology in the left temporal lobe, as revealed by MRI and EEG. Although this finding is intriguing, there is limited attention in the current literature, where laterality is rarely reported. A recent study reported quantitative abnormalities lateralized to the left amygdala in patients with focal epilepsy, even though their epilepsy syndrome did not predominantly localize to the left temporal lobe [36]. The predominance of left‐sided temporal involvement has also been reported in other neuroinflammatory diseases, such as optic neuritis, multiple sclerosis, and also in LGI1 autoimmune encephalitis [37, 38, 39]. Factors such as referral or sampling effects might have biased our cohort toward left temporal predominance. However, the majority of patients in our cohort had generalized, bilateral tonic–clonic seizures irrespective of the affected side defined by LT‐EEG, and the proportion was even higher in the right‐sided subgroup. This suggests that detection bias alone, due to the higher eloquence of the left hemisphere, cannot fully account for the observed pattern, as bilateral tonic–clonic seizures are unlikely to remain unrecognized. Nonetheless, focal seizures arising from the left temporal lobe may be more readily identified in clinical settings because they often present with language impairment and verbal memory deficits.

Contrary to our assumption, MRI measures of microstructural and volume changes showed no correlation with neuropsychiatric symptoms. The facial emotion recognition and the left amygdalar T1w/T2w ratio showed only a weak correlation in our study that did not reach statistical significance (*r* = 0.2). Previous studies have shown that damage to the basolateral amygdala can impact facial emotion recognition [40]. The basolateral complex of the amygdala is integral to fear and anxiety‐related behaviors and emotional facial expressions [41, 42]. Consistent with our findings, a recent study reported no significant associations between microstructural or volumetric MRI measures and neuropsychiatric scores in patients with anti‐NMDA receptor encephalitis [15].

Our volumetric results showed no discernible volume changes of the left or right amygdala in patients with ALE compared to HC. However, exploratory analyses at the nuclei level suggested that the lateral nucleus of the left basolateral complex is swollen in patients with LT ALE. A similar pattern was noted in the other three remaining nuclei of the basolateral complex (basal, accessory basal, and paralaminar) for patients with LT ALE. Prior MRI studies have indicated dynamic changes in amygdala and hippocampus volumes, likely from inflammation and subsequent degeneration and gliosis [9]. A bilateral enlargement of the amygdala was observed in early stages of ALE [9, 10, 43].

This study has several limitations inherent to the investigation of a rare disease. The limited sample size of both patients and HC constitutes a significant limitation of this work and underscores the need for replication in larger cohorts. Moreover, the infrequent occurrence of right temporal ALE precluded statistical analysis of this subgroup. The strength of our study lies in the in‐depth phenotypic characterization of patients using LT‐EEG monitoring at the time of MRI acquisition. This method minimizes the effects of swelling from overt seizure activity on MRI measures. Although antibody heterogeneity may confound some findings, the microstructural changes and the clinical syndrome—including seizures and neuropsychiatric symptoms—were consistent across patients. Another limitation is the lack of systematic neuropsychological testing. The absence of correlations may reflect insufficient power, or limited sensitivity to nuclei‐specific dysfunction, rather than a true lack of association or dissociation between amygdala pathology and neuropsychiatric symptoms. However, while psychiatric symptoms might have been underestimated, the expertise of our multidisciplinary epilepsy clinic reduces the likelihood of missing clinically relevant neuropsychiatric deficits. Most patients were on ASM at the time of MRI, which may have influenced the results. Likewise, the impact of treatment duration and cumulative immunotherapy exposure might have biased some of the findings. However, the majority of patients were treatment‐naïve, and a sensitivity analysis in this subgroup did not alter our results. The cross‐sectional design of this study precludes any temporal inference about histological changes from inflammation, remodeling, and neurogenesis. Therefore, longitudinal imaging is required to study the value of the T1w/T2w ratio as a diagnostic or monitoring biomarker.

In conclusion, our findings indicate a preferential microstructural damage to the basolateral complex of the amygdalar complex in ALE, with a strong lateralization to the left side. These findings correlated with the epileptic focus recorded by LT‐EEG monitoring. This study proposes the clinical utility of the T1w/T2w ratio as a marker for detecting microstructural changes in the amygdala in ALE. Notably, the T1w/T2w ratio can be obtained from standard epilepsy protocols already implemented in the clinical setting, enabling rapid translation into patient care. Using the T1w/T2w ratio could facilitate early and accurate diagnosis of ALE and hence improve treatment outcomes for affected patients.

## Conflicts of Interest

All other authors declare that they have no competing financial interests or personal relationships related to this work.

This work was previously presented as a poster at the Dreiländertagung 2025 conference, held in Salzburg.
